# Determination of anisotropic elastic parameters from morphological parameters of cancellous bone for osteoporotic lumbar spine

**DOI:** 10.1007/s11517-021-02465-0

**Published:** 2021-11-29

**Authors:** Christoph Oefner, Elena Riemer, Kerstin Funke, Michael Werner, Christoph-Eckhard Heyde, Stephan Schoenfelder

**Affiliations:** 1 Faculty of Engineering Sciences, Leipzig University of Applied Sciences, Karl-Liebknecht-Strasse 134, 04277 Leipzig, Germany; 2 Center for Research on Musculoskeletal Systems, Faculty of Medicine, Leipzig University, Semmelweisstrasse 14, 04103 Leipzig, Germany; 3 Department of Orthopaedic Surgery, Traumatology and Plastic Surgery, Leipzig University, Liebigstrasse 18, 04103 Leipzig, Germany; 4 Fraunhofer Institute for Machine Tools and Forming Technology IWU, Nöthnitzer Strasse 44, 01187 Dresden, Germany

**Keywords:** Osteoporotic spine, Cancellous bone, Fabric tensor, Elastic anisotropy, μCT, Homogenization theory

## Abstract

In biomechanics, large finite element models with macroscopic representation of several bones or joints are necessary to analyze implant failure mechanisms. In order to handle large simulation models of human bone, it is crucial to homogenize the trabecular structure regarding the mechanical behavior without losing information about the realistic material properties. Accordingly, morphology and fabric measurements of 60 vertebral cancellous bone samples from three osteoporotic lumbar spines were performed on the basis of X-ray microtomography (μCT) images to determine anisotropic elastic parameters as a function of bone density in the area of pedicle screw anchorage. The fabric tensor was mapped in cubic bone volumes by a 3D mean-intercept-length method. Fabric measurements resulted in a high degree of anisotropy (*DA* = 0.554). For the Young’s and shear moduli as a function of bone volume fraction (BV/TV, bone volume/total volume), an individually fit function was determined and high correlations were found (97.3 ≤ *R*^2^ ≤ 99.1,*p* < 0.005). The results suggest that the mathematical formulation for the relationship between anisotropic elastic constants and BV/TV is applicable to current μCT data of cancellous bone in the osteoporotic lumbar spine. In combination with the obtained results and findings, the developed routine allows determination of elastic constants of osteoporotic lumbar spine. Based on this, the elastic constants determined using homogenization theory can enable efficient investigation of human bone using finite element analysis (FEA).

**Graphical Abstract:**
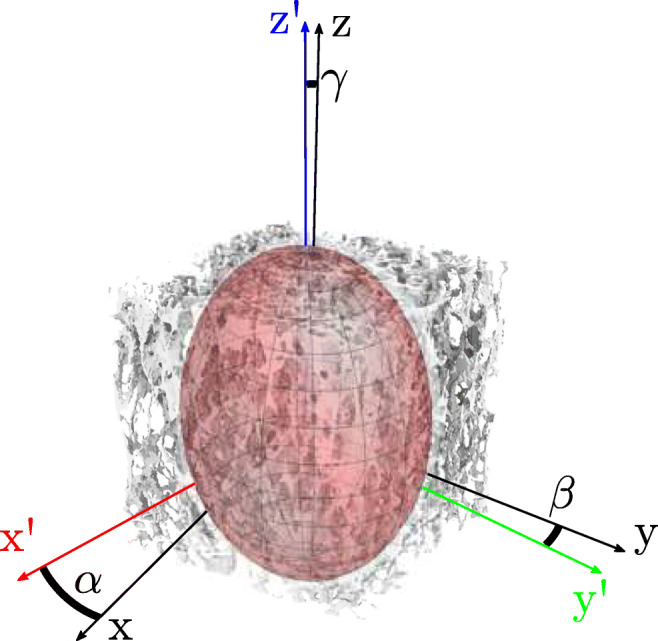
Cancellous Bone with Fabric Tensor Ellipsoid representing anisotropy and principal axis (colored coordinate system) of given trabecular structure

Cancellous Bone with Fabric Tensor Ellipsoid representing anisotropy and principal axis (colored coordinate system) of given trabecular structure

## Introduction

Bone is designed by nature according to the lightweight construction principle and consists of a hard outer layer (cortical bone) and an internal open cell tissue (cancellous bone). The cancellous bone material has highly anisotropic elasticity due to its porous, trabecular structure. Nevertheless, this porous material is responsible for the anchoring of implants in addition to the cortical bone. Due to various factors, such as poor bone quality, inflammation, or short-term implant overloading, the risk of implant loosening or failure increases [33, 44]. In the spine, the pedicle screws loosen in different ways. In osteoporotic bone quality, the risk of loosening is highest [44]. A craniocaudal cutout of the screws can be found primarily in the first weeks after surgery due to cyclic daily stress [33].

Osteoporosis, an imbalance between bone resorption and bone formation, is not only a decrease of the bone mineral density (BMD) but also a change of the morphology of cancellous bone. One of the established bone density measurement methods is the dual-energy X-ray absorptiometry (DXA). The BMD is defined according to the *World Health Organization* (WHO) as the number of standard deviations (SD) above or below the mean for a healthy 30-year-old adult (*T*-score). A low bone mass or osteopenia is defined with − 1 ≤ *T* ≤− 2.5. Osteoporosis is defined as a value for bone mineral density of − 2.5 SD below the young adult mean [23]. Looking at the lumbar vertebrae, the trabeculae become thinner or even disappear resulting in a weakening of the whole structure and bone fractures [29]. Jayasinghe et al. [21] also observed in older and obviously osteoporotic bones that large pores may be present in the vertebral body.

In biomechanics, large numerical models with macroscopic representation of several bones or joints are necessary to answer complex questions. Using finite element analysis (FEA), real material properties of bone are crucial for the best possible assessment of implant loosening mechanisms. Existing and validated material models [20, 43] can predict the post-yield behavior of cancellous bone.

However, implementing these material models in commercial FEA software and applying them to large bone structures remains a major challenge. The complexity of implant-bone systems requires simplifications and suitable methods in simulation. Transferring engineering methods of fatigue life prediction [13, 41] to bones using FEA [14, 36] may provide a means for more efficient calculation. The detailed knowledge of the elastic material parameters of the bone surrounding the screw is crucial for a realistic and valid estimation of the fatigue life of the screw-vertebrae compound. In particular, the use of the FKM guideline [38] in combination with homogenization theory and FEA seems to be a way to predict fatigue behavior of complex bone-implant system.

By using large models, it is not useful to represent and calculate the cancellous bone geometrically. Approaches to homogenize the trabecular structure are necessary without losing information about the realistic material properties and the dependence on bone quality [1, 2, 19, 26, 50]. A first approach has been explored by Cowin [3] using a single second rank tensor (fabric tensor). An alternative model for the anisotropic elasticity based on fabric tensors was presented by Zysset and Curnier [51]. This approach uses a power law relationship, which reduces the number of constants or exponents. Both models are valid, differing only in the complexity and robustness of the fabric-elasticity relationship.

The total stiffness of cancellous bone is a result of the stiffness of the matrix material and the given structure. While the matrix material is nearly isotropic [39], the total stiffness is strongly anisotropic due to trabecular growth, whereby the trabecular structure adapts to the loads present, known as Wolff’s Law [8, 9].

Another challenge for a realistic modeling is the individuality of each person. Due to the different appearing loads in the human body, there are widely varying morphological and material parameters in different bones and also within the bones themselves [10, 27, 31, 47].

These challenges in the field of biomechanics lead to the research aims of this work. First, determination of the morphological indices and elastic constants of human cancellous bone from osteoporotic lumbar spine as a function of bone density based on present X-ray microtomography (μCT) images. Second, analysis of local differences in the lumbar vertebrae with regard to morphological or mechanical parameters in the area of pedicle screw anchorage, respectively. Accurate determination of the elastic constants of osteoporotic bone of the lumbar spine will provide the basis for studies regarding pedicle screw anchorage.

## Materials and methods

### Specimen and imaging

Four fresh-frozen human lumbar spines (1 female, 3 male; L1–L5 levels) stored at − 80^∘^C were available for the examinations. First, DXA measurements were performed to determine the mean BMD value of all specimens (*T*-score, 1st–4th lumbar vertebrae). Second, all 20 lumbar bodies were subjected to a μCT scan (vtomex s 240, GE Sensing & Inspection Technologies GmbH, Germany) with a maximum X-ray tube voltage of 160 kV (500 μA, 68 ms shutter speed). The bone was placed in an airtight sample holder filled with styrofoam and calibration phantoms (water, air, PMMA). No further sample preparation was necessary for the microtomographic imaging.

Based on experience with the thawing and freezing process of human bone and the degeneration that occurs, the resolution was adjusted to prevent thawing during the μCT scan without cooling. A spatial resolution of 80μm was used. The bodies of each lumbar spine were scanned separately and stored in the refrigerator immediately after the scan. Furthermore, a specimen was scanned with a spatial resolution of 20μm to subsequently determine the influence of voxel size on the calculated elastic parameters. The resolution was increased in post-processing calculation step-wise from 20 to 80μm (increment size 20μm) to carry out an error estimation of elasticity parameters from a coarse to a finer resolution.

Imaging, image preparation, and analysis were performed with Fiji [42] and the plug-in BoneJ2 [7]. After an initial examination of the image stacks, the number of samples was reduced due to existing pathological findings (osteoporotic fractures) in a lumbar spine. As a result, image analyses were performed on three male lumbar spines with a median age of 76 years (range from 73 to 85 years). Focusing on pedicle screw anchorage and the aim using the derived results for FEA analyses, the areas for sample preparation were chosen in regions of a conventional pedicle screw trajectory.

**Fig. 1:**
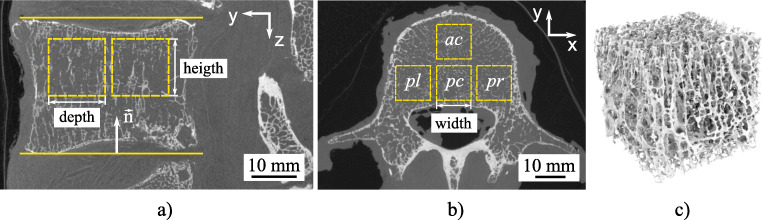
Schematic of sample preparation (a) view on sagittal (yz-)plane with horizontal lines and normal direction, (b) view on transverse (xy-)plane with defined anatomical regions (*a*, anterior; *p*, posterior; *l*, left; *c*, center; *r*, right), (c) example of cubic specimen

Four anatomic regions (Fig. 1b) were defined in the vertebral body, three in the posterior region (left, *pl*; center, *pc*; right, *pr*) and one in the anterior region (*ac*). A cubic shape was selected for the sample geometry to avoid errors due to this in relation to the anisotropic parameters [15]. For each lumbar spine, a local coordinate system was defined, where the z-axis on the end plates is defined in normal direction ($\overrightarrow {n}$). The x- and y-axes are perpendicular to the z-axis in a left-handed coordinate system.

Due to the sagittal spinal profile and the spatial orientation of the lumbar spine in the μCT scan, the vertebral bodies were not aligned in any local principal direction (x, y, z). With an interactive stack rotation in Fiji, the end plates of the vertebral bodies were aligned individually to two parallel horizontal lines (Fig. 1a). To get the cubes in a maximum size, and each perfectly filled with trabecula, the image stack was rotated from the yz- to the xy-plane. Subsequently, four square, non-overlapping areas were manually defined with a side length as a multiple of the spatial resolution. Likewise, attention was paid not to get into the cortical area. In the next step, the image stack was cropped and all images removed including the intervertebral disc or end plate, respectively. Also the image stack was binarized using the isodata algorithm [40] to yield images with pure bone (white) in the foreground and pure marrow (black) in the background.

All samples have different dimensions because the maximum size was selected individually for each area. Furthermore, the areas to be evaluated lie in the superior region of the vertebral bodies, since the pedicle screws are anchored here. With the method described, a total of 60 cubic test specimens were obtained from 15 vertebral bodies (Fig. 1c).

### Morphological parameters

In this examination, the plug-in BoneJ2 was used to determine morphological parameters of the given images of cancellous bone. The plug-in calculates the volume fraction BV/TV, which is the volume of mineralised bone (BV) per unit volume of the sample (TV). It counts all the foreground voxels (bone) and compares them to the total number of voxels in the image. The option bone surface area creates a mesh from the bone in the image, and then calculates the area of the surface of the mesh. To investigate the structure of the tissue also the surface fraction (BS/TV) was determined from the bone surface (BS) and the given total volume of each specimen. The bone surface (BS) area was calculated using surface triangulation of the binary data based on the marching cubes method. Furthermore, for all specimens, the trabecular thickness (Tb.Th.) and trabecular spacing (Tb.Sp.) [17] with mean value, standard deviation (SD), coefficient of variation (CV), median, and minimum and maximum values were evaluated. Equally, the connectivity density (Conn.D.) represents the trabeculae number (Tb.N.) per unit volume and may quantify the bone loss in the trabecular network [35].

The geometry of the individual trabeculae is also of great interest, as the analysis of these can help better predict age- and disease-related fractures [45]. The ellipsoid factor (EF) measures how prolate or oblate the trabecular space is at a particular point. Highly prolate (javelin-shaped, rod-like) ellipsoids have a single long axis (c) and two short axes (a, b) such that *a* < *b* ≪ *c*, whereas highly oblate (discus-shaped, plate-like) ellipsoids have two longer axes (b, c) and one much shorter axis (a) so that *a* ≪ *b* < *c*. Calculating EF as the difference in ratios, EF = *a*/*b* − *b*/*c* leads to a useful scale ranging from − 1 (oblate, *a*/*b* ≈ 0,*b*/*c* ≈ 1) to + 1 (prolate, *a*/*b* ≈ 1,*b*/*c* ≈ 0). EF of 0 indicates an intermediate ellipsoid where *a*/*b* = *b*/*c*, which is the case for spheres (*a* = *b* = *c*) [6].

When considering the surface fraction (BS/TV) of the cancellous bone, it is a physical constraint that bone with no volume (BV/TV = 0) and bone with no pores (BV/TV = 1) have no bone surface (BS/TV = 0). Based on this assumption, a linear relationship between the surface and volume fraction is insufficient. Like Fyhrie et al. [10] has already shown the simplest function with a single unknown parameter (*K*) which satisfies the physical constraints is

1 $$  \mathrm{BS/TV}= K \mathrm{(BV/TV)[1-(BV/TV)]} $$

### Fabric measurement

The fabric of the vertebral cancellous bone samples was measured using the mean intercept length (MIL) [34, 49]. MIL values were determined in 5000 random directions. The MIL, drawn as a radius from the origin under the angle of measurement, generates the surface of an ellipsoid in the three-dimensional cancellous structure. This observation from Harrigan and Mann [15] led to the conclusion that the MIL would be equivalent to a positive definite second rank tensor. Therefore the material anisotropy tensor **M**, whose components in terms of the formula for the ellipsoid are

2 $$  \boldsymbol{M}= \begin{pmatrix} M_{11} & M_{12} & M_{13} \\ M_{21} & M_{22} & M_{23} \\ M_{31} & M_{32} & M_{33} \end{pmatrix} $$

represents the microstructure and anisotropy of cancellous bone. Resulting from the eigenvalues (*D*_1_ ≤ *D*_2_ ≤ *D*_3_) of ***M***, the degree of anisotropy (DA) is calculated as

3 $$ DA= 1 - \frac {D_{1}} {D_{3}} $$

Degree of anisotropy is a measure of how highly oriented substructures are within a volume. Trabecular bone varies its orientation depending on mechanical load and can become anisotropic (DA = 1). The range of 0 < DA < 1 describes anisotropy of various degrees. A value of 0 means the volume is completely isotropic (DA = 0).

Cowin [4] denoted an algebraically related tensor to ***M***, the fabric tensor ***A*** of cancellous bone. ***A*** is related to the material anisotropy tensor ***M*** of Harrigan and Mann [15] by

4 $$ \boldsymbol{A}= \boldsymbol{M}^{-1/2} $$

The three principal dimensions of the ellipsoid represent the eigenvalues *A*_11_, *A*_22_, and *A*_33_ of the fabric tensor, which were sorted such that *A*_11_ > *A*_22_ > *A*_33_. For the fabric measurement of the inhomogeneous vertebral bone the plug-in BoneJ1 was used. The plug-in was adapted due to a necessary modification in the calculation of the fabric tensor ellipsoid’s radii from the eigenvalues, which is confirmed by the author Michael Doube [7]. The fabric eigenvectors ***e***_1_,***e***_2_,***e***_3_ were directly obtained as a result of the fabric tensor in BoneJ1. The three fabric eigenvalues were normalized to

5 $$  \lambda_{i}= \frac {A_{ii}} { A_{11}+A_{22}+A_{33} } \quad (i=1,2,3) $$

which leads to *λ*_1_ + *λ*_2_ + *λ*_3_ = 1.

### Relationship between structural and mechanical anisotropy

Because of the cavities filled with bone marrow between the trabeculae, cancellous bone can be regarded as an inhomogeneous, porous material. A structural and anisotropic material property is created by the principal direction of the trabeculae. The structural anisotropy of the porous material determined with the MIL method and expressed by the fabric tensor has a significant influence on the mechanical properties of trabecular bone. To obtain the mechanical anisotropy, Cowin [3] proposed equations that establish a relationship between the fabric tensor, volume fraction (BV/TV), and the elastic constants. With this homogenization theory of trabecular bone, a porous material can be transformed into a solid with anisotropic material properties.

For an anisotropic linear-elastic material without pre-stress, the following relationship applies:

6 $$  T_{ij}=C_{ijkm}  E_{km} $$

wherein the fourth rank elasticity tensor *C*_*ijkm*_ establishes the relationship between the components *T*_*ij*_ of a stress tensor ***T*** and the components *E*_*km*_ of the strain tensor ***E***. The most general form of the relationship between the elasticity and fabric tensor, consistent with the assumption of an isotropic matrix material *E*_*t*_, is

7 $$  \begin{aligned} C_{ijkm} = {} & E_{t} [a_{1} \delta_{ij} \delta_{km} + a_{2} (A_{ij} \delta_{km} + \delta_{ij} A_{km}) \\ &+ a_{3} (\delta_{ij} A_{kq} A_{qm} + \delta_{km} A_{iq} A_{qj}) \\ &+ b_{1} A_{ij} A_{km}+ b_{2} (A_{ij} A_{kq} A_{qm} + A_{is} A_{sj} A_{km}) \\ &+ b_{3} A_{is} A_{sj} A_{kq} A_{qm} + c_{1} (\delta_{ki} \delta_{mj} + \delta_{mi} \delta_{kj} ) \\ &+ c_{2} (A_{ik} \delta_{mj} + A_{kj} \delta_{mi} + A_{im} \delta_{kj} + A_{mj} \delta_{ki})\\ &+ c_{3} (A_{ir} A_{rk} \delta_{mj} + A_{kr} A_{rj} \delta_{mi} \\ &+ A_{ir} A_{rm} \delta_{kj} + A_{mr} A_{rj} \delta_{ik})] \end{aligned} $$

where *a*_*i*_,*b*_*i*_,*c*_*i*_(*i* = 1,2,3) are functions of BV/TV and *tr****A***,*tr****A***^2^,*tr****A***^3^. There are three indices in Eqs. (6) and (7) and *δ*_*ij*_ is the Kronecker delta.

The representation of the fourth rank elasticity tensor in Eq. (7) is not capable of representing all possible elastic material symmetries. Cowin [3] showed that the least possible symmetry to be represented, expressed by Eq. (7), is orthotropy. Without the presentation of the derivation (see [3]) and taking into account the normalization of the fabric tensor eigenvalues (cf. Eq. (5)), the nine orthotropic elastic constants can be expressed as a function of ***A*** and nine scalar functions of solid volume fraction with

8 $$ \begin{aligned} c_{iiii} = & E_{t} [k_{1} + 2 k_{6} + (k_{2} + 2 k_{7}) \text{II} + 2 (k_{3} + 2k_{8}) \lambda_{i} \\ & + (2 k_{4} + k_{5} + 4 k_{9}) {\lambda_{i}^{2}}] \\ c_{iijj} = & E_{t} \left[k_{1} + k_{2} \text{II} + k_{3} (\lambda_{i} + \lambda_{j}) + k_{4} \left( {\lambda_{i}^{2}} + {\lambda_{j}^{2}}\right) + k_{5} \lambda_{i} \lambda_{j}\right] \\ c_{ijij} = & E_{t} \left[k_{6} + k_{7} \text{II} + k_{8} (\lambda_{i} + \lambda_{j}) + k_{9} \left( {\lambda_{i}^{2}} + {\lambda_{j}^{2}}\right)\right. \end{aligned} $$

where *i*,*j* = 1,2,3;*i*≠*j* and II = *λ*_1_*λ*_2_ + *λ*_1_*λ*_3_ + *λ*_2_*λ*_3_. The nine functions of volume fraction (*k*_1_ − *k*_9_) were calculated with

9 $$  k_{i} = k_{i\mathrm{a}} + k_{i\mathrm{b}} (\mathrm{BV/TV})^{p} $$

with 18 constants *k*_*ia*_ and *k*_*ib*_ (cf. Table 1). The isotropic tissue modulus of the pure matrix material has been defined as *E*_*t*_ = 15GPa [39].

**Table 1:** Used constants *k*_*ia*_ and *k*_*ib*_ from [22] with an adjusted squared correlation coefficient ($R_{adj}^{2}$= 95.6%) as a goodness-of-fit criterion [46]

*i*	*k*_*ia*_	*k*_*ib*_
1	− 6.523 ⋅ 10^− 3^	6.238 ⋅ 10^− 1^
2	2.758 ⋅ 10^− 1^	1.157 ⋅ 10^1^
3	− 2.631 ⋅ 10^− 1^	− 1.343 ⋅ 10^1^
4	2.629 ⋅ 10^− 1^	− 1.159 ⋅ 10^1^
5	2.739 ⋅ 10^− 1^	1.879 ⋅ 10^1^
6	1.782 ⋅ 10^− 2^	1.206 ⋅ 10^− 1^
7	− 7.129 ⋅ 10^− 2^	− 8.538
8	3.820 ⋅ 10^− 3^	− 8.001
9	5.276 ⋅ 10^− 3^	− 1.051 ⋅ 10^1^
*p*	1.6	

**Fig. 2:**
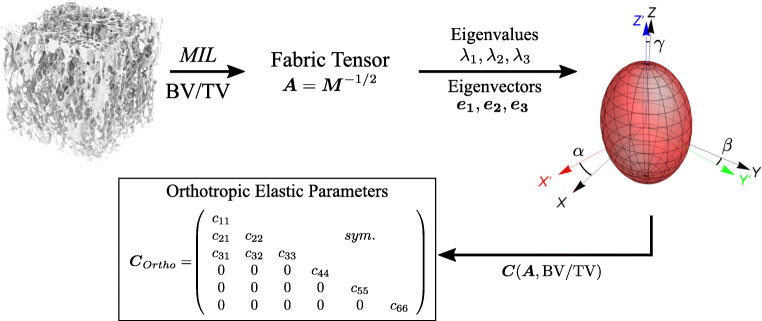
Methodical procedure for obtaining orthotropic elastic parameters as a function of bone volume fraction (BV/TV) from cancellous bone by μCT images, *MIL* and fabric tensor

In this work, the constants *k*_1_ − *k*_9_ and the exponent *p* were used from Kabel et al. [22] (cf. Table 1). They covered six different anatomic sites (calcaneus, proximal tibia, distal femur, proximal femur, proximal humerus, and lumbar vertebral bodies) and calculated the best fit between fabric and elastic variables. Despite the correlation from different anatomical regions in their work, the applicability to the lumbar spine is assumed here. Thereupon, Kabel et al. [22] used the goodness of each fit evaluated by correlating elastic coefficients obtained from finite element analysis to elastic coefficients predicted from the analytical material models of continuum mechanics. Based on the methods and equations presented, a routine (Fig. 2) for calculating the anisotropic constants was created. Calculations were performed using Wolfram Mathematica 11.1.

As shown in Fig. 2, the MIL ellipsoid, whose principal directions are defined by the three eigenvectors (***e***_***1***_,***e***_***2***_,***e***_***3***_), will be potentially rotated (x’, y’, z’) in three-dimensional space to the main coordinate system (x, y, z). The occurring angles (*α*,β,*γ*) between the respective main principal axis and the principal axis of the ellipsoid were calculated by means of directional cosine. As a result from Eqs. (2) to (9), the orthotropic stiffness tensor ***C***_Ortho_ in Voigt notation can be derived as a function of fabric tensor ***A*** and the volume fraction BV/TV. On the mathematical relation ***C***^− 1^ = ***S*** between the stiffness tensor ***C*** and the compliance tensor ***S*** the elastic parameters, the Young’s modulus *E*, the shear modulus *G*, and the Poisson ratio *ν* can be calculated. These parameters are often determined in uniaxial tests, like the uniaxial compression test, and used for the description of the stiffness behavior of bone. The Young’s modulus *E* is defined for the uniaxial stress state in the principal direction *i* as

10 $$  E_{i} = \frac {1}{S_{ii}},  i=1,2,3 $$

The shear modulus *G* is similarly determined in the direction *i* to

11 $$  G_{i} = \frac {1}{S_{ii}},  i=4,5,6 $$

The Poisson ratio *ν* as a parameter for the strain of a material in perpendicular direction (*j*) to the direction of load (*i*) for *i*≠*j* is calculated as follows:

12 $$  \nu_{ij} = - E_{i} S_{ij} = - \frac {S_{ij}}{S_{ii}},  i=1,2,3 $$

The symmetry of the stress and strain tensors indicates that not all six Poisson ratios in Eq. (12) are independent. The remaining three Poisson ratios being derived from the relations

13 $$  \frac {\nu_{21}}{E_{2}} = \frac {\nu_{12}}{E_{1}}, \frac {\nu_{31}}{E_{3}} = \frac {\nu_{13}}{E_{1}}, \frac {\nu_{23}}{E_{2}} = \frac {\nu_{32}}{E_{3}} $$

## Results

With a mean *T*-score of − 3.2 specimen 2 (85 years) showed the lowest value, followed by − 2.9 (specimen 1, 73 years) and − 2.3 (specimen 3, 76 years). The extracted and examined cubic specimens from the vertebral bodies had a side length of 12.75mm ± 0.99mm (mean ± SD) resulting in a cubic volume of 2106.3m*m*^3^ ± 457.5m*m*^3^.

The results of the morphological and fabric measurements are shown in Table 2. The volume fraction BV/TV ranges from 8.2 % to 19.4 %. Both the largest (L5-*pc*) and the lowest (L2-*pl*) bone volume and surface fractions were found in specimen 3. While the bone volume and surface fractions have constant values within a specimen, the results of parameters trabecular thickness (Tb.Th.), trabecular spacing (Tb.Sp.), and ellipsoid factor (EF) are based on individual specimen mean values. The mean values of each sample evaluated in BoneJ2 were used for mean value and SD of the specimen. In specimen 3, the lowest (L2-*pr*) and highest (L3-*pr*) trabecular thickness were present. The largest cavities expressed by Tb.Sp. were found in specimen 1 (L5-*pc*), and the smallest in specimen 2 (L3-*pr*).

**Table 2:** Descriptive statistics of the morphological and fabric data

Index	Mean	SD	CV(%)	Median	Min.	Max.
BV/TV (−)	0.133	0.024	17.70	0.131	0.082	0.194
BS/TV (m*m*^− 1^)	1.677	0.228	13.58	1.694	1.141	2.264
Tb.Th. (μm)	260.7*	29.5*	11.30*	252.4*	219.3*	373.1*
Tb.Sp. (μm)	984.4*	132.1*	13.42*	969.3*	774.5*	1294.6*
EF (−)	0.064*	0.027*	42.30*	0.064*	-0.821	0.979
Conn.D. (m*m*^− 1^)	1.50	0.54	35.79	1.50	0.12	2.87
DA (−)	0.554	0.067	12.00	0.559	0.365	0.694
*D*_1_ (−)	0.326	0.114	34.90	0.337	0.112	0.543
*D*_2_ (−)	0.617	0.201	32.54	0.625	0.225	1.118
*D*_3_ (−)	0.731	0.238	32.54	0.723	0.252	1.297

^*^ Values based on individually specimen mean values from BoneJ2 over all 60 samples

The largest scatter (CV = 42.30 *%*) in the results showed the ellipsoid factor, i.e., plate-like (EF=− 0.821) and rod-like (EF= 0.979) trabeculae could be determined in the vertebral cancellous bone. The wide range between the values that occurred resulted in a mean value of approximately zero. A connectivity density of 1.50m*m*^− 1^ ± 0.54m*m*^− 1^ indicates approximately 1.5 connected trabeculae in the osteoporotic cancellous bone per unit volume (1m*m*^3^).

An average degree of anisotropy of DA = 0.554 shows that the longest principal dimension of the fabric ellipsoid is twice the size of the shortest dimension. The cancellous bone architecture is oriented within the sample and accordingly anisotropic in a range from DA = 0.365 to DA = 0.694. The eigenvalues (*D*_1_ − *D*_3_) of the material stiffness anisotropy tensor from the ellipsoidal approximation are also provided in Table 2.

**Fig. 3:**
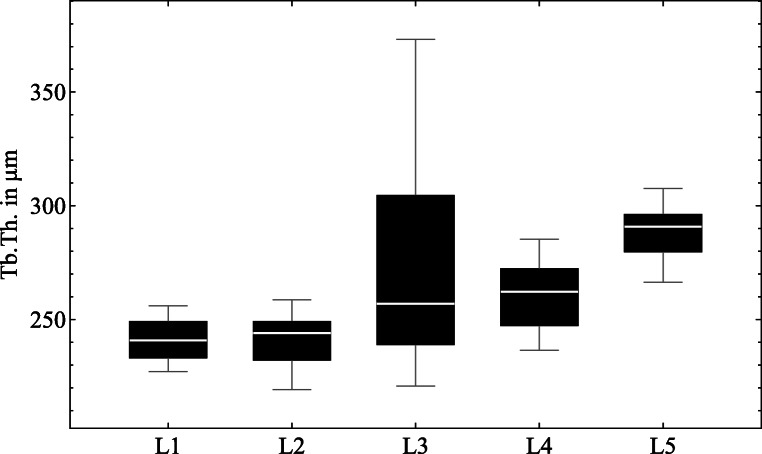
Boxplot of all Tb.Th. values (in μm) of cancellous bone over the five lumbar vertebrae (L1 - L5)

In order to see which indices are related to bone density or anatomical regions, further evaluations were carried out. Figure 3 shows the trabecular thickness in a box plot for the five lumbar bodies. It can be recognized that the trabeculae become thicker in the caudal direction with a slight increase of the mean values from 242μm (L1) to 288μm (L5).

**Fig. 4:**
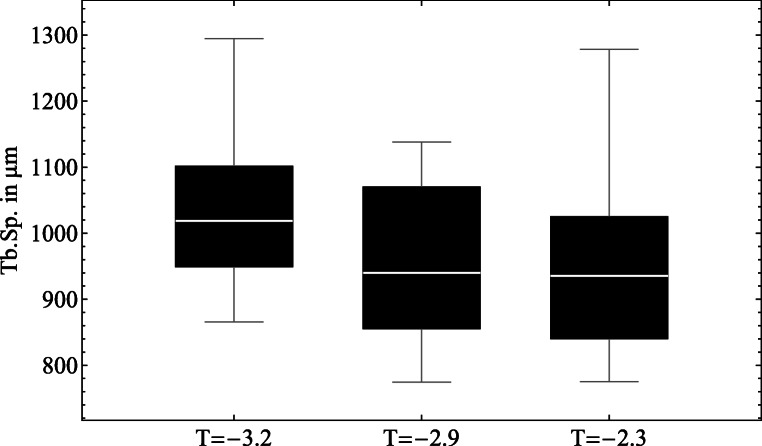
Boxplot of all Tb.Sp. values (in μm) from cancellous bone plotted over the three donors with measured *T*-score

Expecting a relation between the trabecular spacing and *T*-score, the mean values (Tb.Sp.) of all samples were assigned to the particular lumbar spine *T*-scores and shown in a box plot (Fig. 4). A significant decreasing pore size (Kruskal-Wallis test, *p* < 0.05) is recognizable with increasing bone density within the three donors.

**Fig. 5:**
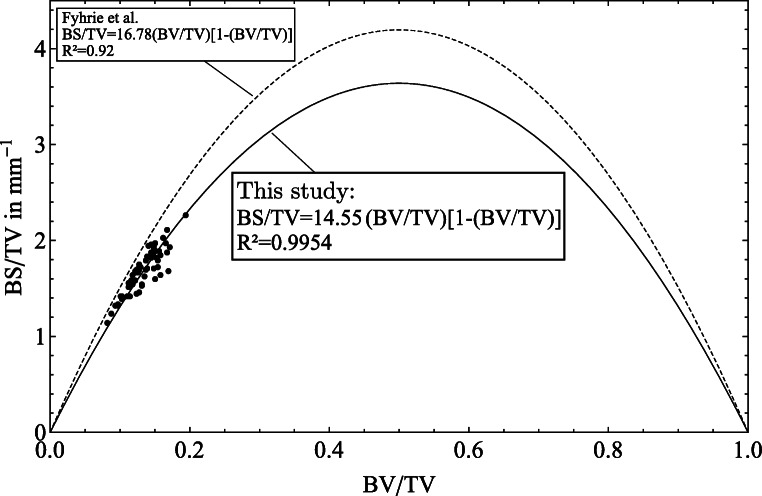
The total bone surface per total volume (BS/TV) predicted by the bone volume fraction (BV/TV) using the model: *y* = *Kx*(1 − *x*)

Based on the results of surface fraction (BS/TV) from all 60 samples, a prediction by the volume fraction using a quadratic function (K = 14.55,*R*^2^ = 99.54*%*) could be carried out as shown in Fig. 5.

**Fig. 6:**
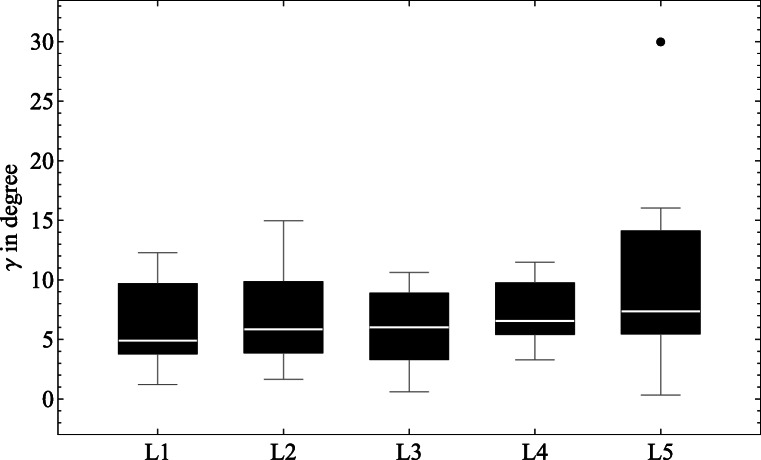
Boxplot showing the primary orientation (*γ*) of the trabeculae in the lumbar vertebrae

The angle *γ* (see Fig. 2, Chapter 2.4) in the five individual lumbar vertebral bodies (L1–L5) was evaluated as an indicator for the orientation of the trabeculae to the main coordinate system. By definition and resulting from measurements *γ* lies not perfectly in the sagittal plane. Nevertheless, it is a good indication of the principal orientation of trabeculae to the normal direction ($\overrightarrow {n}$) or z-axis, respectively. With a median value of *γ* = 4.9^∘^ in the specimens for L1, a small increase to *γ* = 7.4^∘^ in the specimens for L5 can be seen (Fig. 6). There are large standard deviations and a relative high positive skewness (except L3) in this result. In all specimens, the angle *γ* was measured in a range from 0.6^∘^ to 16.0^∘^ with an outlier of 30.1^∘^.

The results of the evaluation of how highly the trabeculae are orientated in the various defined anatomical regions within a lumbar vertebra are shown in Table 3. It is noticeable that the trabeculae appear to be more rotated toward the end plate where the pedicles enter the vertebral body than in the other central regions.

**Table 3:** Orientation of trabeculae in different anatomic regions

Region	*γ*[^∘^](mean ±SD)
*ac*	5.99 ± 4.29
*pc*	5.61 ± 3.71
*pl*	9.85 ± 6.62
*pr*	7.56 ± 3.32

**Fig. 7:**
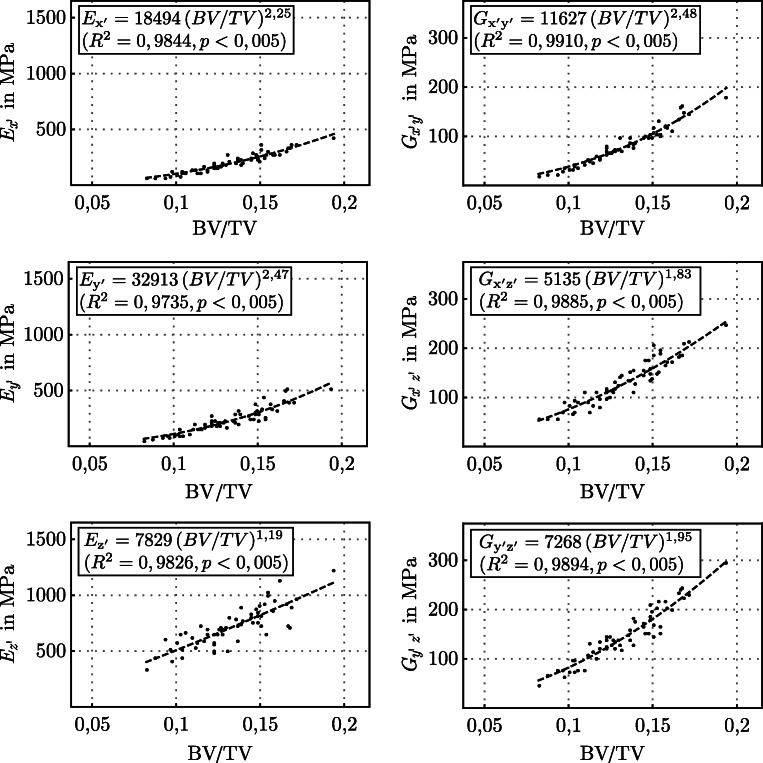
Calculated Young’s and shear moduli as a function of bone volume fraction (BV/TV). Correlation of elastic parameters to volume fraction (dotted lines) yields in individually fit function using the model: *y* = *ax*^*b*^

As a major aim of this study, a correlation of the nine orthotropic elastic parameters to the bone density expressed by volume fraction BV/TV is elaborated. Figure 7 shows the relationship for the Young’s and shear moduli in each principal direction with $i=1,2,3 \hat {=} x^{\prime },y^{\prime },z^{\prime }$. Assuming a linear elastic solid with a volume fraction of BV/TV= 0, i.e., cancellous bone without solid bone and pure marrow, theoretically the material cannot resist deformation in response to an applied force. For this reason, the results were fitted in each direction to a function in the general form *y* = *ax*^*b*^.

Using the existing relationship, the nine orthotropic elastic constants can be expressed as a function of ***A*** and nine scalar functions of solid volume fraction. For every elastic parameter, an individually fit function (dotted lines) was determined. Most remarkable are high correlations (0.9735 ≤ *R*^2^ ≤ 0.9910) with a high significance (*p* < 0.005) for the Young and shear moduli.

It can be seen that the highest Young’s modulus is found in the z’-axis, followed by the y’- and x’-axes, depending on the bone volume fraction. The highest shear modulus occurs correspondingly in the y’-z’ plane, with the parameters depending on the bone volume fraction. For the Poisson ratios, non-significant or very weak correlations were found. Nevertheless, the Poisson ratios are consistent with the current Young’s and shear moduli for an orthotropic material and lie within a range of 0.039 < *ν* < 0.510. All values of Young’s and shear moduli were normalized to the isotropic tissue modulus *E*_*t*_ (cf. Table 4).

**Table 4:** Descriptive statistics of the homogenized orthotropic elastic constants normalized to the unit bone tissue modulus *E*_*t*_ = 15GPa

Variable	Mean	SD	Min.	Max.
$E_{x^{\prime }}/E_{t}$	13.6 ⋅ 10^− 3^	5.73 ⋅ 10^− 3^	3.69 ⋅ 10^− 3^	27.70 ⋅ 10^− 3^
$E_{y^{\prime }}/E_{t}$	15.76 ⋅ 10^− 3^	7.41 ⋅ 10^− 3^	3.02 ⋅ 10^− 3^	34.50 ⋅ 10^− 3^
$E_{z^{\prime }}/E_{t}$	47.60 ⋅ 10^− 3^	11.86 ⋅ 10^− 3^	22.31 ⋅ 10^− 3^	81.44 ⋅ 10^− 3^
$G_{y^{\prime }z^{\prime }}/E_{t}$	9.80 ⋅ 10^− 3^	3.49 ⋅ 10^− 3^	3.09 ⋅ 10^− 3^	19.68 ⋅ 10^− 3^
$G_{x^{\prime }z^{\prime }}/E_{t}$	8.70 ⋅ 10^− 3^	2.96 ⋅ 10^− 3^	3.69 ⋅ 10^− 3^	16.36 ⋅ 10^− 3^
$G_{x^{\prime }y^{\prime }}/E_{t}$	5.46 ⋅ 10^− 3^	2.46 ⋅ 10^− 3^	1.17 ⋅ 10^− 3^	11.92 ⋅ 10^− 3^
$\nu _{y^{\prime }z^{\prime }}$	0.109	0.030	0.056	0.186
$\nu _{x^{\prime }z^{\prime }}$	0.098	0.025	0.039	0.160
$\nu _{x^{\prime }y^{\prime }}$	0.300	0.058	0.202	0.464
$\nu _{z^{\prime }y^{\prime }}$	0.352	0.050	0.257	0.510
$\nu _{z^{\prime }x^{\prime }}$	0.358	0.034	0.296	0.471
$\nu _{y^{\prime }x^{\prime }}$	0.337	0.047	0.233	0.454

**Fig. 8:**
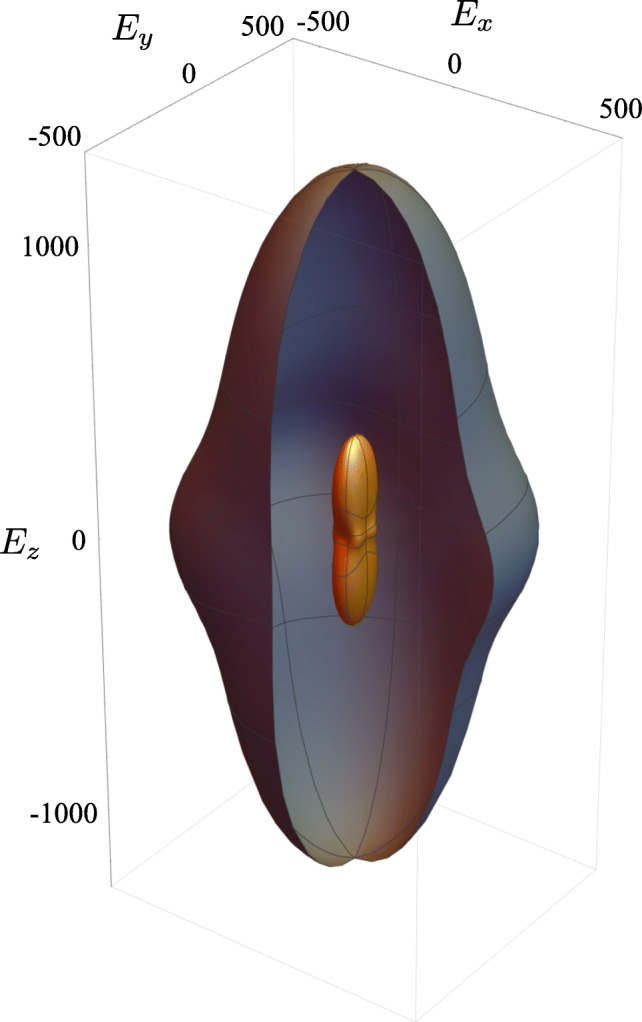
Visualization of directional dependent Young’s moduli in three-dimensional space for the highest (gray) and the lowest (orange) bone volume fraction. For a better comparability of the maximum stiffness ratio the outer surface was cut open. Values in MPa

In fact, the elasticity tensor calculated by homogenization theory can be visualized by using a symmetric fourth rank tensor. A surface is created by rotating the fourth rank tensor in three-dimensional space, whose shape can be interpreted as the Young’s modulus for an elongation test in the particular direction in space. Both for the largest (L5-*pc*) and the smallest (L2-*pl*) bone volume fractions (specimen 3), the directional-dependent Young moduli were visualized as surface and compared (cf. Fig. 8). The shape for different volume fraction is similar, but the dimensions corresponding to the stiffness vary significantly.

### Error estimation of determined elastic parameters

**Fig. 9:**
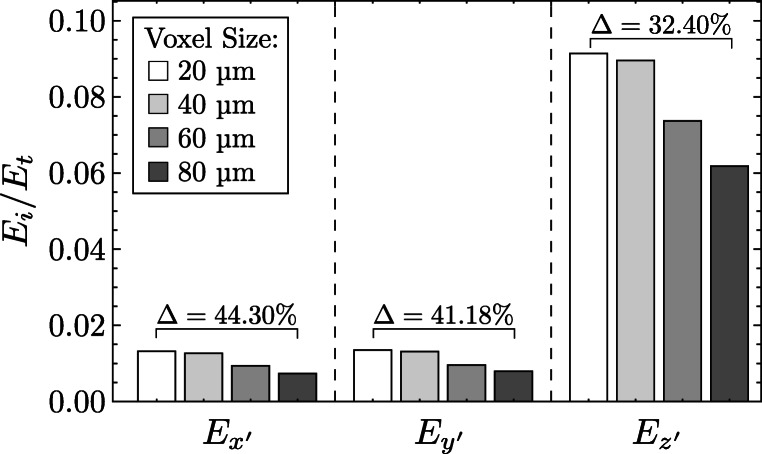
Normalized Young’s moduli in dependence on voxel size (original voxel size: 20 *μ* m)

Since the voxel size could not be reduced due to the necessary further use of the bone samples for mechanical examinations (thawing of the samples should be avoided), an error estimation on the voxel size was performed. The determination of the influence of voxel size on elastic parameters showed that as voxel size increases, the stiffness of cancellous bone determined by homogenization theory decreases. This relationship is illustrated in Fig. 9 using the example of the dependence of the determined Young’s moduli on the voxel size.

The elastic properties showed a greater sensitivity on the voxel size in the x’- and y’-directions than in the z’-direction. With increasing voxel size (increment 20 μm), the determined stiffness of the cancellous bone decreases. In direct comparison between the original voxel size (20 μm) from the μCT scan and the artificially generated resolution of 80 μm for the error estimation, the relative error was determined. Those amounts to 44.30 % (*E*_*x*_), 41.18 % (*E*_*y*_), and 32.40 % (*E*_*z*_) for the individual components. Similar relative errors and the same trend of lower stiffness with increasing voxel size could be obtained for the shear moduli as a function of voxel size.

## Discussion

It was the aim in this work to calculate morphological indices and elastic constants of human cancellous bone from osteoporotic lumbar spine and to determine the resulting bone elastic constants as a function of bone density from μCT images. For these purposes, we have calculated morphological indices and elastic constants for a total of 60 bone samples from three specimens. Additionally, differences in morphological and mechanical parameters within a human vertebral body were investigated. Various regions were defined and evaluated for this purpose.

### Imaging and morphology

BMD measurement and evaluation using DXA are currently the gold standard for diagnosing osteoporosis. However, BMD measurements do not fully account for changes in trabecular architecture, tissue properties, and accumulation of microdamage [29]. Osteoporosis can also lead to minor compression fractures in the lumbar area or calcification is present in elderly people. These diseases can induce errors in the estimation of lumbar BMD and falsely higher values [30]. This phenomenon appears to have occurred in one donor in this study (female, 94 years, *T* = − 1.6); thus, these samples were excluded. The three donors finally examined showed bone quality in the osteoporotic and strongly osteopenic regions.

The resolution of μCT images used in the present study (80 μm) is higher than presently can be obtained in vivo. Based on a resolution study, Müller et al. [32] showed a high dependency of morphological indices on resolution. They concluded that reconstructions with a resolution of about 175 μm are sufficient and the values restorable when using calibration procedures. However, with the trabecular thickness in this range, a finer resolution seems reasonable.

The morphological indices measured with the image analysis software Fiji from lumbar osteoporotic spines showed a good agreement to other studies [10, 12, 16, 22, 27, 37, 47]. With values of 8.2*%* < BV/TV < 19.5*%* the bone volume fraction was found in a physiological range for osteoporotic lumbar spine [37]. No significant relations between Tb.Th. or Tb.Sp. and BV/TV were found, which can be explained by very poor values and the small range of volume fraction [47]. Also, the four different regions defined within a vertebral body may have caused a large dispersion of morphological indices. For example, both the highest and the lowest BV/TV and BS/TV were present in the same donor (specimen 3).

Anatomical circumstances like venae basis vertebralis, growth of pedicle, or local defects have a great influence on relative small sample sizes (mean side length: 12.75mm). The basivertebral veins become greatly enlarged in advanced age [11], which can be confirmed by our observations. This may affect morphological parameters in the central posterior region (*pc*), where the point of exit of the basivertebral vein is located. Otherwise, no implant anchorage is necessary in this region and the influence is negligible.

While there is a very weak correlation between individual bone volume fractions and trabecular thickness (Tb.Th.) or trabecular spacing (Tb.Sp.), global relationships have been found. The trabeculae become thicker in the caudal direction, which corresponds to the greater loads on the spinal column in this direction. The differences between the donors and the parameter Tb.Sp. are also significant (*p* < 0.05), which is a clear indication of deteriorating bone quality with a decreasing *T*-score.

Another structural index for declining bone quality is the surface fraction. A high correlation (*K* = 14.55,*R*^2^ = 99.54*%*) between the surface and volume fraction based on a nonlinear regression analysis (Fig. 5) was found compared to *K* = 16.78 (*R*^2^ = 0.92) from Fyhrie et al. [10]. With a mean value of BS/TV = 1.677m*m*^− 1^ the results are comparable to Fyhrie et al. [10] and Hildebrand et al. [16]. The results of Ulrich et al. [47] with an average of BS/TV = 23.37m*m*^− 1^ for the lumbar spine are controversial based on our results.

The geometry of vertebral cancellous bone showed a mainly plate-like character. Nevertheless, also rod-like trabeculae were found in the bone samples. The results agree with the observations in lumbar vertebrae of Jayasinghe et al. [21]: “In individuals with substantial porosity, the remaining vertical elements are plates, forming the walls of large tubular spaces that run mainly in the longitudinal axis of the bone. The remaining bone tissue is reduced to thin rod-like elements running predominantly in the horizontal axis of the bone”.

### Fabric measurement and elastic constants

Fabric measurements were performed using MIL based on the findings of Kabel et al. [22] that MIL is the best model for predicting elastic properties. The analysis was based on the relationships developed by Cowin [3] assuming equal tissue elastic modulus and nine functions of volume fraction. The use of these functions from literature [22] is feasible because elastic properties of cancellous bone from fracture patients can be estimated from morphological parameters [18]. In agreement with earlier studies [24, 46, 48], a function of volume fraction to the power of *p* = 1.6 was used, which lies within the known interval between 0.5 < *p* < 3. The nine functions of volume fraction were determined for a smaller region of interest and a different scanner resolution (20–25 μm) by Kabel et al. [22]. As shown in Fig. 9, different voxel sizes can significantly affect the results. The use and transfer to larger region of interest applied here may additionally affect the results.

This study shows that the anisotropic elastic properties of cancellous bone in the osteoporotic lumbar spine are to be calculated from architectural parameters and volume fraction. With a systematic analysis of the morphology and stiffness of 60 bone samples from the osteoporotic lumbar spine between 97.3 and 99.1*%* of the anisotropic parameters were explained by BV/TV and fabric measurements. While Ulrich et al. [47] could explain only 37 − 67*%* of the variance of elastic constants for human trabecular bone by BV/TV, also Maquer et al. [27] showed that 97*%* of the mechanical stiffness of cancellous bone can be explained by BV/TV and fabric tensor.

The highest stiffness was determined in z’-direction which can be explained by Wolff’s law and the adaption of trabeculae to the highest loads. The Young’s moduli for the y’- and x’-axis are quite similar, with a slightly larger stiffness in the y’-direction. Also, Ulrich et al. [47] and Gudde [12] found comparable results. Extrapolating the fit function to a volume fraction of BV/TV = 1, which would correspond to a solid, non-porous material, results in a Young’s modulus of $E_{z^{\prime }}=17.45 \text {GPa}$. With a defined isotropic tissue modulus of 15GPa, the results seem to overestimate the theoretically possible stiffness in that direction. For the vertical directions (x’,y’), other effects seem to have an influence on the resulting stiffness which are approximately reduced by factor 3 (BV/TV= 1). The applied formulae [3] in combination with nine functions of volume fraction and the MIL method constitute a much lower stiffness.

In this study, Poisson’s ratios exceed a known value for solid material of *ν*_*ij*_ = 0.5. This is permissible in anisotropic materials. Open cell materials like hexagonal honeycombs can exhibit Poisson’s ratio of 1 [25]. Indeed, comparable results to our study were found for orthotropic materials like wood [28] and cancellous bone [47]. A large part of the derived elastic parameters are valid for a volume fraction lower than 15 %. As proposed by Parkinson et al. [37], spontaneous fractures below a threshold of BV/TV = 15*%* for vertebral bodies can occur more frequently.

The evaluation of the orientation of the trabeculae to the end plates showed an orientation of the trabecular structure that increased from 6.2^∘^ (L1) to 10.0^∘^ (L5). These results are comparable to Gudde [12], who also found an increase from L1 (5.4^∘^) to L5 (7.0^∘^) in the lumbar vertebrae. Another study from Damasceno et al. [5] supports our results. There the intervertebral disc angles were measured in the lumbar spine to evaluate the sagittal profile. The results presented were arising from 5.0^∘^ (L1) to 12.3^∘^ (L5) which support the concept that the principal direction of trabeculae seems to follow the lordotic posture of the lumbar spine.

The error determination in Fig. 9 showed that a systematic error results from the voxel size. Thus, the values of elastic constants should be used with caution in a quantitative manner, taking into account the error. Nevertheless, the relationships between the individual elastic constants remain in the respective direction, so that a qualitative relationship can be assumed. Using the values of the elastic parameters determined in this work with a voxel size of 80 μm, for example in finite element analysis, would represent a conservative assumption. In uniaxial compression tests, a higher stiffness of the bone would have to be expected in the specific direction than calculated with homogenization theory and fabric tensor. For a quick use of the elastic parameters in dependence of the bone volume fraction, e.g., in FEA, the best fit functions from Fig. 7 are recommended.

### Limitations

There are some limitations in this study. First, μCT scanning cannot currently be performed in vivo on humans due to excessive radiation dose. Therefore, the method shown remains in the research environment being to improve our understanding of the effects of changes in bone quality and to understand implant failure. Until it is used as a clinical instrument for the diagnosis of bone quality further research is required in the field of imaging devices. Second, the range of bone volume fraction was small based on the specimens available. Therefore, the determined correlations and statistical validation between bone volume fraction and elastic parameters are limited to a small range. Third, no μFE models were created from the original images, which means that no evidence can be provided that the calculated homogenized properties correspond to the values predicted by μFE models. Fourth, the resolution of the images was relatively coarse for the current state of the art in μCT imaging. Unwanted thawing of the specimens significantly limited the scan time and thus resolution.

## Conclusion

From the results obtained, the following findings can be briefly summarized as follows:

- The mathematical formulation for the relationship between anisotropic elastic constants of a porous, inhomogeneous material and bone volume fraction is applicable to the conducted and used μCT data of cancellous bone in osteoporotic lumbar spine.
- The relationship between anisotropic elastic parameters and the bone volume fraction of osteoporotic cancellous bone determined here can be used directly in FEA, e.g., to study implant loosening mechanisms by predicting fatigue life.
- Within a lumbar vertebra, large local differences in the mechanical properties of cancellous bone were detected. For the use of the anisotropic properties in FEA, a multi-zone definition of material parameters in the area of implant anchorage is therefore proposed.
- The principal direction of trabeculae follows the lordotic posture of the lumbar spine. Similarly, the greatest stiffness is present in the longitudinal direction, i.e., it follows the main load direction that occurs, known by Wolff’s law.

The major strength of this work are the methods used and the results obtained allow a local determination of the elastic material properties for the osteoporotic lumbar spine. Evaluations of the anatomic conditions also confirmed the understanding of osteoporotic cancellous bone in the literature.

## Declarations

All procedures performed in studies involving human participants were in accordance with the ethical standards of our institutional (Institute for Anatomy, University Leipzig) and with the 1964 Helsinki declaration and its later amendments or comparable ethical standards. The body donor program is regulated by the Saxonian Death and Funeral Act of 1994 (third section, paragraph 18, item 8).
